# Universal, School-Based Mental Health Program Implemented Among Racially and Ethnically Diverse Youth Yields Equitable Outcomes: Building Resilience for Healthy Kids

**DOI:** 10.1007/s10597-023-01090-5

**Published:** 2023-02-09

**Authors:** Jessica L. Chandrasekhar, Anne E. Bowen, Erin Heberlein, Emily Pyle, Christina R. Studts, Stacey L. Simon, Lauren Shomaker, Jill L. Kaar

**Affiliations:** 1 Division of Endocrinology, Department of Pediatrics, University of Colorado School of Medicine, Aurora, CO USA; 2 Children’s Hospital Colorado, Aurora, CO USA; 3 Children’s Hospital Colorado Colorado Springs, Colorado Springs, CO USA; 4 Department of Human Development and Family Studies, Colorado State University, Fort Collins, CO USA

**Keywords:** Adolescence, School, Coaching, Resilience, Equity

## Abstract

Although suicide is a leading cause of mortality among racial and ethnic minority youth, limited data exists regarding the impact of school-based mental health interventions on these populations, specifically. A single-arm pragmatic trial design was utilized to evaluate the equity of outcomes of the universal, school-based mental health coaching intervention, Building Resilience for Healthy Kids. All sixth-grade students at an urban middle school were invited to participate. Students attended six weekly sessions with a health coach discussing goal setting and other resilience strategies. 285 students (86%) participated with 252 (88%) completing both pre- and post-intervention surveys. Students were a mean age of 11.4 years with 55% identifying as girls, 69% as White, 13% as a racial minority, and 18% as Hispanic. Racial minority students exhibited greater improvements in personal and total resilience compared to White students, controlling for baseline scores.

## Introduction

Suicide represents a major preventable cause of death among youth. In the United States, suicide deaths among individuals aged 5–18 years increased nearly 70% from 2001 to 2018 ("Web-based Fatal Injury Data Visualization Tool"). The greatest increase was present among adolescents aged 10–14 years, among whom the rate has more than doubled. Similarly, the prevalence of suicide ideation also has increased rapidly among adolescents, and one-third of youth endorsing suicide ideation will eventually attempt suicide (Nock et al., 2013). Cha et al. (2018) review the epidemiology and potential risk factors for youth suicide ideation and attempt (i.e., “suicide thoughts and behaviors”). In particular, low self-esteem/negative self-reference (Burke et al., 2016; Soto-Sanz et al., 2019), loneliness (Gallagher et al., 2014; Zygo et al., 2019), relational conflicts (Randell et al., 2006; Zygo et al., 2019), and mood/anxiety disorders (Séguin et al., 2011) have been linked to suicide thoughts and behaviors among youth. By contrast, “resilience,” i.e., exhibiting positive adaptation when faced with adversity, instead may represent a protective process against suicide thoughts and behaviors (Felver et al., 2019; Hornor, 2017; Lee et al., 2012; Luthar & Cicchetti, 2000). To address the major public health issue of adolescent suicide, intervening to promote resilience during the transition to early adolescence—a period notable for the increased risk for suicide thoughts and behaviors—holds promise.

Certain racial and ethnic minority populations in the United States are linked with a particularly high prevalence of suicide thoughts and behaviors. Native American youth have the highest rate of suicide deaths across all racial and ethnic groups ("Web-based Fatal Injury Data Visualization Tool"). Black children aged 5–11 years have a suicide death rate that is more than double that of White children ("Web-based Fatal Injury Data Visualization Tool"). Likewise, according to the Youth Risk Behavior Survey (YRBS) conducted in 2019, a greater percentage of non-Hispanic Black high school students reported attempting suicide compared to Hispanic and non-Hispanic White students (Ivey-Stephenson et al., 2020). In general, the rate of suicide attempts by Black youth has been increasing (Bridge et al., 2015; Ivey-Stephenson et al., 2020; Lindsey et al., 2019). By contrast, the 2019 YRBS results demonstrated a decreased rate of suicide attempts by Hispanic students since 2015; however, a greater percentage of Hispanic girls attempted suicide compared to non-Hispanic White girls, and overall suicide attempt rates have increased among Hispanic adolescents since the previous survey in 2017 (Ivey-Stephenson et al., 2020). Factors that may underlie such disparities in suicide thoughts and behaviors include acculturation pressures/stress (Silva & Van Orden, 2018), intergenerational trauma (Wexler et al., 2016), peer victimization (Geoffroy et al., 2016; Winsper et al., 2012), and discrimination (Sellers et al., 2006; Xu et al., 2020). Ultimately, there is a critical need for resilience-focused interventions that are proven effective among racial and ethnic minorities, specifically.

The existing literature highlights a role for school-based mental health interventions in bolstering youth resilience, in part given the opportunity for widespread application (Kuperminc et al., 2020; Lee et al., 2012; Seligman et al., 2009). A meta-analysis by Dray et al. (2017) demonstrates the effectiveness of universal, resilience-focused programs in decreasing psychological distress, symptoms of depression, and internalizing and externalizing problems. Likewise, an early analysis of Building Resilience for Healthy Kids (i.e., Healthy Kids) found significant increases in resilience and self-efficacy (Lee et al., 2020). Healthy Kids is a universal, school-based intervention, utilizing weekly 1:1 sessions between health coaches and students to promote resilience. Notably, while Healthy Kids and similar programs have demonstrated overall improvements for participants, few studies have examined intervention outcomes specifically among racial and ethnic minorities.

The intent of this study was to determine whether Healthy Kids, as a universal intervention without specific cultural adaptations, can be appropriately implemented in a racially and ethnically diverse population, considering the unique barriers and needs that racial and ethnic minority populations may face. Through this focus, we hoped specifically to address the gap in the existing literature surrounding equitable school-based mental health interventions. We expected that Healthy Kids would demonstrate similar improvements among students identifying as non-Hispanic White, Hispanic, or a non-Hispanic racial minority (Black, Native Hawaiian/Pacific Islander, Asian, American Indian, more than one race) given the highly individualized approach of the intervention which allowed for personalized goals and related coaching for each student.

## Methods

### Study Design and Intervention

This study took place at an urban public middle school in Colorado Springs, Colorado from January-March 2020. School selection was based on an expressed interest in partnership and a critically high level of youth suicide thoughts and behaviors in the region, surpassing that of the state average ("Healthy Kids Colorado Survey (2019): Mental Health"). For ethical reasons given this local rate of suicide thoughts and behaviors, a single-arm pragmatic trial study design intentionally was utilized instead of a randomized controlled trial. Therefore, all students enrolled in the sixth grade as of January 2020 were invited to participate in Healthy Kids. Based on school-reported demographics, 71% of the total students enrolled at the middle school are White, 15% are Hispanic, and 16% qualify for free/reduced cost meals.

Healthy Kids is a universal, school-based, resilience-focused intervention that has previously been described in detail (Lee et al., 2020). Briefly, students attend an initial 1:1 session with a health coach to build rapport and provide an overview of the program. Students then are scheduled to meet weekly with that same coach for six weeks. These 15-min intervention sessions incorporate motivational interviewing techniques and focus on personalized goal setting and strategies for improving resilience, in areas such as coping and developing positive relationships. After the final week, the health coach and student meet to summarize progress and to discuss opportunities for continued growth. This intervention was developed based on the framework created by the Center on the Developing Child at Harvard University ("Key Concepts: Resilience") and the 7 “C”s of resilience established by the American Academy of Pediatrics (Ginsburg & Jablow, 2005).

### Cultural Humility Training

Many authors have highlighted the need for cultural awareness and humility in youth mental health interventions (Lee & Wong, 2020; Pumariega et al., 2005; Scott et al., 2015; Weisz et al., 2005). The interactions of each coach-student dyad within Healthy Kids likely are influenced by the diverse personal beliefs, values, and background that each brings to the encounter. Therefore, all Healthy Kids coaches received cultural humility training comprising an initial self-assessment, didactic information, and a dedicated group discussion centered around these materials. This training was intended to increase general awareness and to spark an interest in lifelong growth among coaches related to the importance of culture in influencing an individual’s experience within the program and society, more broadly.

### Key Measures

Online surveys were completed by participants at school using the REDCap platform. A pre-intervention survey was administered in January 2020, and a post-intervention survey was administered in March 2020, immediately following the last session. The main outcome measures for this study assessed personal, relationship-based, and total resilience. Measures of academic pressure, grit, depression and anxiety symptoms, and social, emotional, academic, and total self-efficacy were secondary outcomes.

### Demographics and Health Behaviors

Age, gender, race, and ethnicity were reported by the school based on parent-provided school registration data.

### Mental Health Parameters

*Resilience* was measured via the Child and Youth Resilience Measure using Rasch analysis (CYRM-R). The 17-item CYRM-R assesses i) personal resilience— internal factors that convey an individual’s ability to respond to negative emotions and events; and ii) relationship-based resilience— the perceived availability of supportive relationships. Respondents rate each item from “not at all” to “a lot” using a 5-point scale, and sub-scores for personal and relationship-based resilience are summed to obtain a total resilience score. Higher scores suggest greater resilience. The CYRM-R has been validated for youth aged 11–19 years with adequate internal reliability (Cronbach’s α = 0.82) (Jefferies et al., 2019).

*Self-efficacy* is comprised of: i) social self-efficacy— a perceived capability to build and nurture interpersonal relationships and to express assertiveness; ii) academic self-efficacy— a perceived capability to meet academic expectations and to achieve scholastic mastery; and iii) emotional self-efficacy— a perceived capability to manage and overcome negative emotions. The 24-item Self-Efficacy Questionnaire for Children (SEQ-C) was utilized to obtain sub-scores for each of these components, which then were summed to yield a total score. In this tool, each item is rated using a 5-point scale ranging from “not at all” to “very well,” and higher scores suggest greater self-efficacy. The SEQ-C has been validated for youth aged 12–19 years with adequate internal reliability (α = 0.90) (Muris, 2002).

*Grit* is a non-cognitive trait comprised of general perseverance and a drive towards long-term goals. It was evaluated using the 12-item Grit Scale, wherein each item is measured on a 5-point scale ranging from “not at all like me” to “very much like me.” Higher scores suggest more grit. The 12-item Grit Scale has been validated for youth aged 7–15 years with adequate internal reliability (α = 0.80) (Duckworth et al., 2007).

*Academic pressure* was measured using the 16-item Educational Stress Scale for Adolescents (ESSA). Each item in this tool is rated from “strongly disagree” to “strongly agree” using a 5-point scale, and higher scores suggest more academic pressure. The ESSA has been validated for youth aged 12–18 years with adequate internal reliability (α = 0.81) (Sun et al., 2011).

*Anxiety and depression symptoms* were evaluated using the Patient-Reported Outcomes Measurement Information System (PROMIS) Pediatric Anxiety and Depressive Symptoms Scales, which assess mood over the past 7 days. Each scale includes 8 items measured on 5-point scales ranging from “never” to “almost always,” and higher scores suggest more elevated symptoms of anxiety and depression. These scales have been validated for youth aged 8–17 years with adequate internal reliability (α = 0.85) (Irwin et al., 2010).

### Statistical Analysis

Analyses were conducted using SAS version 9.4 (SAS institute, Inc, Cary NC). Preliminary analyses using independent samples t-tests demonstrated no significant differences between individual racial minority populations (Black, Native Hawaiian/Pacific Islander, Asian, American Indian, more than one race) for pre- and post-intervention data. Therefore, we aggregated those students into a larger “non-Hispanic racial minority” grouping to allow for further analyses. Linear regressions were used to analyze differences by race and ethnicity (non-Hispanic White, non-Hispanic racial minority, and Hispanic) for pre-intervention, post-intervention, and change in mental health parameters while controlling for the baseline value of the given parameter. Hedges’ *g* effect sizes were calculated for pre- to post-intervention changes in each of the mental health parameters. Hedges’ *g* corrects for overestimations of the true population effect present in Cohen’s *d*. Interpretations of the size of the effect are consistent with those for Cohen’s *d* (small: 0.2; medium: 0.5; large: 0.8) (Cohen, 1988). Alpha for the present study was set at 0.05 for two-sided tests.

## Results

In January 2020, 285 students (86%) enrolled in the program out of a total of 330 sixth-grade students. The most commonly reported reason for why students/parents opted out was due to missing class time for the coaching sessions. Among those enrolled, 252 students (88%) completed both pre- and post-intervention surveys and were included in these analyses. There were no statistically significant differences between those included in the analysis and those removed (n = 33) by age, race/ethnicity, and gender.

Of the students completing both surveys, 55% were girls, and the mean age was 11.4 years (± 0.5) (Table 1). Based on school registration data, 69% of these students were non-Hispanic White, 13% were a non-Hispanic racial minority (Black, Native Hawaiian/Pacific Islander, Asian, American Indian, more than one race), and 18% were Hispanic.

**Table 1:** Demographic characteristics of students

	*N* = *252*
Gender	
Boys	114 (45%)
Girls	138 (55%)
Age (years)	11.4 ± 0.5
Race/Ethnicity	
Non-Hispanic White	173 (69%)
Non-Hispanic Racial Minority	34 (13%)
Black	11 (32%)
Native Hawaiian/Pacific Islander	4 (12%)
Asian	12 (35%)
American Indian	2 (6%)
More than one race	5 (15%)
Hispanic	45 (18%)

### Baseline

At baseline, there were significant differences by race and ethnicity for total (p = 0.001), social (p = 0.004), emotional (p = 0.01), and academic self-efficacy (p = 0.004) (Table 2). For each of these types of self-efficacy, non-Hispanic racial minority and White students reported higher levels than Hispanic students. There were no significant differences for resilience, grit, academic pressure, and depression and anxiety symptoms.

**Table 2:** Mental health characteristics of students, stratified by race and ethnicity

	White	Racial Minority	Hispanic	*p-value*
	n = 173	n = 34	n = 45	
Pre-intervention				
Resilience	75 ± 6.8	77 ± 5.7	77 ± 7.7	0.10
Personal	44 ± 4.5	43 ± 5.1	44 ± 4.2	0.33
Relationship	32 ± 3.2	31 ± 3.4	32 ± 2.6	0.42
Grit	3 ± 0.5	3 ± 0.5	3 ± 0.4	0.47
Self-efficacy	**82 ± 15**	**83 ± 15**	**72 ± 15**	**0.001**
Social	**28 ± 5.7**	**28 ± 6.2**	**25 ± 6.2**	**0.004**
Emotional	**26 ± 5.8**	**26 ± 5.7**	**23 ± 6.0**	**0.01**
Academic	**28 ± 5.9**	**28 ± 6.0**	**25 ± 5.5**	**0.004**
Academic pressure	40 ± 14	43 ± 13	45 ± 13	0.07
Mood symptoms				
Depression	15 ± 8.4	15 ± 7.5	18 ± 9.5	0.19
Anxiety	18 ± 7.9	18 ± 7.7	19 ± 8.6	0.88
Post-intervention				
Resilience	77 ± 6.9	80 ± 7.0	77 ± 6.0	0.06
Personal	**45 ± 4.4**	**47 ± 4.5**	**44 ± 4.7**	**0.03**
Relationship	33 ± 3.2	33 ± 2.9	33 ± 2.2	0.27
Grit	3 ± 0.5	3 ± 0.5	3 ± 0.4	0.51
Self-efficacy	92 ± 17	90 ± 18	84 ± 21	0.07
Social	31 ± 6.2	31 ± 5.3	28 ± 7.2	0.08
Emotional	28 ± 6.6	28 ± 7.1	26 ± 8.2	0.29
Academic	31 ± 6.3	30 ± 7.2	29 ± 6.6	0.22
Academic pressure	40 ± 13	43 ± 13	45 ± 15	0.10
Mood symptoms				
Depression	15 ± 8.0	14 ± 7.2	17 ± 9.2	0.28
Anxiety	18 ± 7.7	17 ± 7.6	19 ± 8.9	0.33

Bold text indicates significant values, as determined by one-way ANOVA; alpha set at 0.05. Values are included as: avg ± stdev

### Pre- to post-intervention

Linear regression analyses were used to compare changes from pre- to post-intervention, controlling for baseline scores (Table 3). Non-Hispanic racial minority students exhibited greater improvement in total [β = 2.9 (95%CI 0.3, 5.5); p = 0.03] and personal [β = 2.2 (95%CI 0.6, 3.8); p = 0.01] resilience compared to non-Hispanic White students. Non-Hispanic racial minority students also demonstrated greater improvement in total [β = 3.2 (95%CI 0.3, 6.2); p = 0.03] and personal [β = 2.9 (95%CI 0.8, 4.9); p = 0.01] resilience compared to Hispanic students. Significant post-intervention differences in personal resilience were present by race and ethnicity, with non-Hispanic racial minority students displaying greater personal resilience than Hispanic and non-Hispanic White students (p = 0.03) (Table 2).

**Table 3:** Linear regression of mental health characteristics, stratified by race and ethnicity

	White	Racial Minority	*p-value*	Hispanic	*p-value*	Racial Minority (Ref: Hispanic)	*p-value*
	n = 173	n = 34		n = 45		n = 34	
Resilience	Ref	**2.9 (0.3, 5.5)**	**0.03**	− 0.4 (− 2.6, 1.9)	0.74	**3.2 (0.3, 6.2)**	**0.03**
Personal	Ref	**2.2 (0.6, 3.8)**	**0.01**	− 0.5 (− 2.0, 0.9)	0.49	**2.9 (0.8, 4.9)**	**0.01**
Relationship	Ref	1.1 (0.0, 2.2)	0.06	0.3 (− 0.6, 1.3)	0.50	0.7 (− 0.5, 1.9)	0.23
Grit	Ref	− 0.1 (− 0.2, 0.1)	0.29	0.1 (− 0.3, 0.2)	0.12	− 0.2 (− 0.3, 0.0)	0.08
Self− Efficacy	Ref	− 0.7 (− 6.6, 5.3)	0.83	− 0.1 (− 5.5, 5.3)	0.98	− 0.7 (− 9.8, 8.4)	0.88
Social	Ref	0.3 (− 1.8, 2.3)	0.79	0.3 (− 1.3, 1.9)	0.67	0.3 (− 2.0, 2.6)	0.81
Emotional	Ref	0.5 (− 1.3, 2.3)	0.59	0.3 (− 1.5, 2.1)	0.76	− 0.1 (− 3.2, 3.0)	0.96
Academic	Ref Ref	− 1.2 (− 3.1, 0.6)	0.19	0.1 (− 1.6, 1.9)	0.88	− 1.7 (− 4.4, 1.1)	0.23
Academic pressure	Ref	0.1 (− 3.2, 3.3)	0.97	1.4 (− 1.9, 4.6)	0.41	− 1.2 (− 5.9, 3.5)	0.61
Mood Symptoms							
Depression	Ref	− 0.3 (− 2.2, 1.6)	0.76	0.3 (− 1.6, 2.2)	0.77	− 0.8 (− 3.6, 2.1)	0.61
Anxiety	Ref	− 1.0 (− 3.0, 1.0)	0.32	1.1 (− 1.0, 3.1)	0.30	− 2.1 (− 5.1, 0.9)	0.17

Bold text indicates significant values, as determined by linear regression controlling for baseline scores; alpha set at 0.05. Values are included as: β (95% CI)

No significant differences in pre- to post-intervention changes were found by race or ethnicity for the parameters of relationship-based resilience, grit, self-efficacy, academic pressure, and mood symptoms, controlling for baseline scores (Table 3). However, while participants differed significantly by race and ethnicity on baseline measures of self-efficacy, pre- to post-intervention changes in self-efficacy were similar across these groups. No significant post-intervention differences by race and ethnicity were seen in self-efficacy scores (Table 2).

**Fig. 1:**
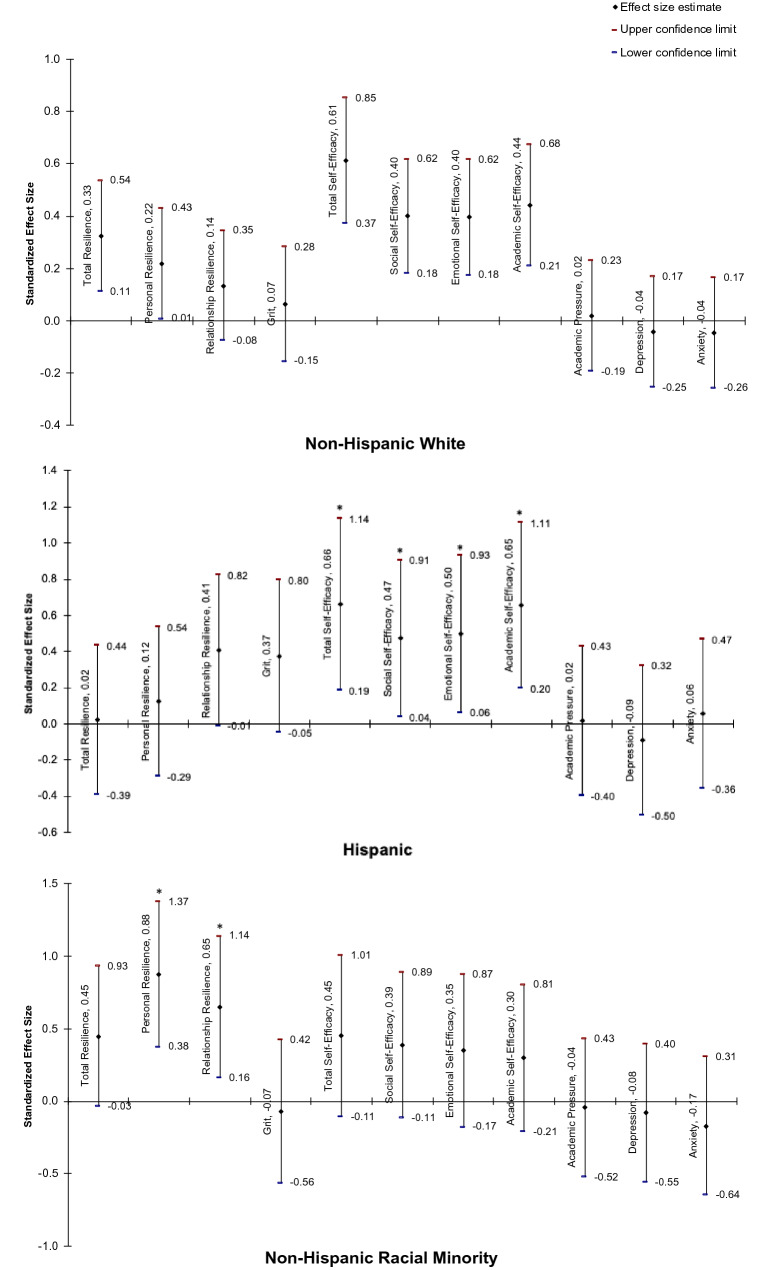
Healthy Kids intervention effect size, stratified by race and ethnicity

Hedges’ *g* effect sizes were calculated with stratification by reported race and ethnicity. Non-Hispanic White students exhibited small to medium effect sizes for total resilience (*g* = 0.33; 95%CI 0.11, 0.54; p < 0.001), personal resilience (*g* = 0.22; 95%CI 0.01, 0.43; p = 0.04), total self-efficacy (*g* = 0.61; 95%CI 0.37, 0.85; p < 0.001), social self-efficacy (*g* = 0.40; 95%CI 0.18, 0.62; p < 0.001), emotional self-efficacy (*g* = 0.40; 95%CI 0.18, 0.62; p < 0.001), and academic self-efficacy (*g* = 0.44; 95%CI 0.21, 0.68; p < 0.001) (Fig. 1). Non-Hispanic racial minority students demonstrated medium to large effect sizes for personal resilience (*g* = 0.89; 95%CI 0.38, 1.37; p < 0.001) and relationship-based resilience (*g* = 0.66; 95%CI 0.16, 1.14; p = 0.01). Hispanic students displayed medium effect sizes for total self-efficacy (*g* = 0.66; 95%CI 0.19, 1.14; p = 0.01), social self-efficacy (*g* = 0.47; 95%CI 0.04, 0.91; p = 0.03), emotional self-efficacy (*g* = 0.50; 95%CI 0.06, 0.93; p = 0.02), and academic self-efficacy (*g* = 0.65; 95%CI 0.20, 1.11; p < 0.001).

## Discussion

Building Resilience for Healthy Kids was associated with significantly larger improvements in resilience among adolescents identifying as Black, Native Hawaiian/Pacific Islander, Asian, American Indian, or more than one race compared to those identifying as non-Hispanic White or Hispanic, despite similar baseline resilience scores. Additionally, although non-Hispanic racial minority and White students reported significantly higher levels of each type of self-efficacy at baseline than Hispanic students, all students reported improved self-efficacy post-intervention and differences by race and ethnicity were no longer detected. While our single-arm study design precludes conclusions about causality, these results are promising in that the Healthy Kids intervention may be associated with improved resilience particularly among racial minority students, as well as with increases in self-efficacy to similar levels among racial and ethnic groups, despite baseline differences for Hispanic students. By the previously discussed increased risk for suicide thoughts and behaviors in these populations (i.e., racial minority and Hispanic), these findings thus suggest potential equity of the therapeutic outcomes for Healthy Kids.

To our knowledge, limited data exists regarding race- and ethnicity-based differences in psychological outcomes following the implementation of universal, school-based, resilience-focused interventions in the United States. A previous study by Ijadi-Maghsoodi and colleagues (2017) demonstrated improved personal resilience when their intervention, Resilience Classroom Curriculum, was implemented among primarily racial and ethnic minority youth. Similarly, Felver and colleagues (2019) examined the effect of a mindfulness-based intervention, Learning to BREATHE, with a racially and ethnically diverse sample of adolescents. Learning to BREATHE potentially conveyed “protective-stabilizing factors,” as the control group demonstrated decreased resilience from pre- to post-intervention while the intervention group remained stable. However, neither of these studies specifically examined outcomes by race and ethnicity.

Our results suggest that Healthy Kids may be an equitable intervention option. Mental health disparities were present at baseline, but, following intervention, psychological measures were similar across racial and ethnic identities. Likewise, Healthy Kids produced greater improvements in resilience among non-Hispanic racial minority students, an early adolescent population which is at a high risk for suicide thoughts and behaviors. The current data demonstrate the potential for universal school-based programs that emphasize individualized coaching, allowing each coach-student dyad to utilize the intervention in a way that is most effective for them. However, the specific underlying reasons for this observed improvement in resilience among non-Hispanic racial minority students can only be theorized at this time and thus present an opportunity for future research.

Disparities in access to and/or utilization of mental health resources are well-documented (Alegria et al., 2010; Costello et al., 2014). Such disparities may explain the greater improvement to resilience seen among racial minority youth in our intervention, as Healthy Kids may represent a source of accessible support at a critical time within the psychosocial development of early adolescence. Alternatively, or complementarily, the health coaches may fill a mentor role. Van Dam et al. (2018) highlight numerous positive youth outcomes associated with non-parent adult mentors. Likewise, a meta-analysis by Chu et al. (2010) specifically identifies that social support from school personnel has the strongest association with child and adolescent well-being, as defined by academic achievement, psychological adjustment, self-concept, and parent–child relationships. Further research is necessary to determine whether the Healthy Kids coach-student relationship itself explains the increased resilience especially present among racial minority youth in this study.

A key strength of this study is that it places a primary focus on evaluating outcomes among racial and ethnic minority populations that are often inadequately assessed during school-based mental health interventions. This study puts forth important data suggesting that universal programs, even without specific intentional cultural adaptation, can demonstrate equal and even equitable improvements among these racial and ethnic minority youth when individualized coaching and goal-setting approaches and cultural humility are appropriately emphasized. Healthy Kids, at its core, relies on a goal-oriented, and thus growth- or asset-based, individualized approach. In general, it is important to highlight and foster an individual’s unique existing strengths to most effectively promote positive outcomes (Luthar & Cicchetti, 2000).

Nonetheless, several limitations must be considered in interpreting these results. Our pragmatic single-arm pre-post design was selected in response to ethical considerations in the context of the aforementioned high rates of adolescent suicide in the students’ community, but such an analysis precludes attributions of changes in resilience, self-efficacy, and other outcomes to the intervention given the potential for confounding variables. Additionally, the racial and ethnic stratification used in these analyses represents an oversimplified categorization in the setting of the robust and heterogenous cultures belonging to individual racial/ethnic minority populations. While no significant differences were seen between those students included in the combined non-Hispanic racial minority group, as mentioned above, subgroup analyses for early adolescents identifying as Black, Native Hawaiian/Pacific Islander, Asian, American Indian, or more than one race would lend further support for the results described here. For the purpose of this specific study, we combined these populations to facilitate further analyses, as these populations, more generally, are at an increased risk for suicide thoughts and behaviors. Finally, our study was unable to assess intersecting points of social identity, such as between race/ethnicity and gender identity and sexual orientation. Girls (Ivey-Stephenson et al., 2020) and youth identifying as lesbian, gay, bisexual, and/or transgender (Almeida et al., 2009; Hatzenbuehler, 2011; Ivey-Stephenson et al., 2020) have higher rates of suicide thoughts and behaviors. Building upon this preliminary work, future studies should utilize randomized controlled trial designs with larger and similarly, or ideally more, diverse samples. Such trials would allow the effectiveness of Healthy Kids to be tested in the context of multiple points of intersectionality among identity components (e.g., race, ethnicity, sex, gender identity, and sexual orientation).

## Conclusions

Racial and ethnic identities may be associated with shared sociopolitical experiences, such as discrimination and marginalization, that can lead to disparities in mental health outcomes. Indeed, an increased prevalence of suicide thoughts and behaviors has been demonstrated among youth who identify as a racial and/or ethnic minority. As a result, it is critical that we empirically confirm that we are offering effective mental health interventions for these youth, specifically. Building Resilience for Healthy Kids may represent an equitable and accessible option for improving resilience and self-efficacy among racial minority and Hispanic youth, respectively. From a public health standpoint, we recommend that Healthy Kids and other mental health interventions are widely implemented in schools given the current suicide epidemic among youth. From a clinical standpoint, clinical pediatric programs (i.e., medicine, psychology, social work, etc.) should foster partnerships with local schools and promote and assist with these programs, particularly encouraging those that have demonstrated effectiveness in racial and ethnic minority populations, specifically. Health professionals, researchers, and policy makers together must advocate for improved training in and incorporation of equitable, culturally aware, and individualized interventions that best support these racial and ethnic minority youth who are at a particularly high risk for suicide thoughts and behaviors.
