# Correction to: Universal, School-Based Mental Health Program Implemented among Racially and Ethnically Diverse Youth Yields Equitable Outcomes: Building Resilience for Healthy Kids

**DOI:** 10.1007/s10597-023-01114-0

**Published:** 2023-03-16

**Authors:** Jessica L. Chandrasekhar, Anne E. Bowen, Erin Heberlein, Emily Pyle, Christina R. Studts, Stacey L. Simon, Lauren Shomaker, Jill L. Kaar

**Affiliations:** 1 Division of Endocrinology, Department of Pediatrics, University of Colorado School of Medicine, Aurora, CO USA; 2 Children’s Hospital Colorado, Aurora, CO USA; 3 Children’s Hospital Colorado Colorado Springs, Colorado Springs, CO USA; 4 Department of Human Development and Family Studies, Colorado State University, Fort Collins, CO USA

**Correction to: Community Mental Health Journal**

10.1007/s10597-023-01090-5

Due to an error during the production process, the original version of this article erroneously included two tables unrelated to the text and a reference to those tables. These have now been removed and the original article has been updated.

